# BODY IMAGE PERCEPTION AND DIETING AMONG “SCREENAGERS” IN POLAND

**DOI:** 10.13075/ijomeh.1896.02643

**Published:** 2025

**Authors:** Anna Dzielska, Joanna Mazur, Katarzyna Lewtak, Jaroslava Kopcakova, Dorota Kleszczewska

**Affiliations:** 1 Institute of Mother and Child, Department of Child and Adolescent Health, Warsaw, Poland; 2 University of Zielona Gora, Collegium Medicum, Department of Humanization in Medicine and Sexology, Zielona Góra, Poland; 3 Medical University of Warsaw, Department of Social Medicine and Public Health, Warsaw, Poland; 4 P.J. Safarik University, Medical Education Centre, Faculty of Medicine, Košice, Slovakia; 5 Palacky University Olomouc, Olomouc University Social Health Institute, Olomouc, Czechia; 6 Institute of Mother and Child, Department of Health Sociology, Education and Medical Communication, Warsaw, Poland

**Keywords:** BMI, adolescents, body image perception, screen time, weight reduction behavior, screen-use profiles

## Abstract

**Objectives:**

This study analyses screen-use profiles among Polish adolescents and their relationship to body image and weight reduction behavior. The authors aimed to identify different screen-use profiles, determine the prevalence of these profiles in the population, and explore the relationships between these screen time profiles, adolescents' body image, and weight reduction behavior.

**Material and Methods:**

The analysis drew upon data from 5322 students aged 13 years, 15 years, and 17 years who participated in the 2021/2022 Health Behaviour in School-aged Children (HBSC) study. The HBSC questionnaire contains data on various screen-related activities, including gaming, social media usage, internet browsing, and the consumption of audiovisual material, as well as their body image perception and dieting behavior. Using the k-means clustering method, 5 distinct screenuse profiles were identified.

**Results:**

The following screen-use profiles were identified: moderate users (40.1%), game-oriented users (21.1%), social-media-oriented users (20.3%), intensive game and social media users (11.1%), and intensive users (7.4%). Girls predominated in the social media clusters, while boys were overrepresented in the gaming clusters. Adolescents in the 2 intensive-use profiles (intensive game and social media users and overall intensive users) exhibited a more than twofold increase in the likelihood of perceiving themselves to be fat and engaging in dieting practices, even after adjusting for BMI, in comparison with students classified in other groups. A higher BMI Z-score independently predicted both body dissatisfaction (OR = 2.36) and active dieting (OR = 2.67).

**Conclusions:**

A significant association was found between screen use patterns and both body image perceptions and dieting behaviors among adolescents in the intensive-use groups. This finding highlights the profound psychosocial impact of all forms of screen-based media and underscore the need for targeted interventions promoting media literacy and healthy digital habits, especially among older adolescents, girls, and urban youth.

## Highlights

- Five screen-use profiles were found among Polish adolescents.
- Intensive screen use is associated with body dissatisfaction and dieting.
- Social-media clusters are dominated by girls, gaming clusters by boys.
- Screen-use profiles vary by age, gender and place of residence.
- Media literacy should address screen-time-related body concerns.

## INTRODUCTION

This study focuses on contemporary adolescents, often referred to as “screenagers”, a term that combines “screen” and “teenagers” to describe the generation now growing up in a media-saturated environment [1]. As a result of rapid technological advancements and changes in daily life, young people today are spending a substantial, and growing, amount of time using electronic devices [2,3]. Routine intensive engagement with various screen-based technologies for communication, education and entertainment, not only shapes their habits and lifestyles but also has significant implications for their health and wellbeing. There are potential benefits of certain activities, such as playing games, using social media or searching for information online, in a variety of areas, like supporting the development of cognitive skills [4], fostering social relationships or providing equal access to educational content, especially for young people living in areas with limited resources [5]. Computer games can support problem-solving and strategic thinking, while social media helps young people to maintain peer relationships and gain emotional support, especially in situations where opportunities for offline contact are limited [6]. Conversely, increased screen time has been associated with poorer physical health and a greater prevalence of psychosomatic complaints [7]. It has also been linked to increased risk of mental health problems, including heightened stress and depressive symptoms [8] and to developmental challenges in emotional and social functioning, such as diminished face-to-face interactions and impaired emotional comprehension [9]. Furthermore, prolonged screen time may be associated with reduced physical activity and poor eating habits [10]. Additionally, it has been correlated with aggressive behavior in children and adolescents [11] and may negatively impact sleep duration and quality [12].

Adolescence is a critical period for body image development, marked by increased sensitivity to social influences and self-evaluation [13]. Excessive screen time, particularly on social media, has been linked to body dissatisfaction in adolescents [14], often manifesting as feeling too overweight or too thin, and can lead to weight-control behaviors or dieting intentions [15]. These effects can be understood in terms of several different theoretical models. For example, social comparison theory, originally proposed by Festinger in 1954 [16], posits that adolescents are especially prone to upward comparisons, evaluating themselves against idealized and often unrealistic portrayals in digital media, which may intensify body dissatisfaction [16,17]. Moreover, sociocultural pressure and internalization of thin ideals can trigger negative affect and restrictive eating behaviors [18]. Repeated exposure to unrealistic appearance content may lead to the internalization of thin or fit ideals, which increases the risk of body dissatisfaction and dieting behaviors [19]. Moreover, the widespread presence of fitness- and body-related trends (e.g., “fitspiration” or “body goals”) reinforces a sociocultural climate where weight control is normalized and often portrayed as a form of self-discipline or success. Exposure to screen-based media heightens these risks, especially during adolescence, a phase of identity formation and heightened sensitivity to appearance-related feedback [19–21]. For adolescents navigating identity development and social belonging, this can translate into heightened pressure to conform to these ideals through dieting, even in the absence of medical necessity.

A recently published international comparative report presenting data from the Health Behaviour in School-Aged Children (HBSC) survey, comparing data from adolescents in 44 countries across Europe and beyond [22], points to a worrying increase in negative body weight perception across age groups and genders, with an increasing number of adolescents perceiving themselves as overweight. Nearly one-third of surveyed adolescents (29%) considered themselves to be “too fat.” Girls were more likely than boys to report this perception, and the gender gap widened with age. The lowest prevalence was observed among 11- and 15-year-old boys (23%), while the highest was found among 15-year-old girls (38%). Alarmingly, Polish adolescents ranked first among their peers from all surveyed countries in all 3 age groups in terms of perceiving themselves as “too fat.”

There is, therefore, a growing need for a more detailed examination of the Polish adolescent population, in particular, in relation to the link between screen time behaviors and body image perception. A separate international study by Dzielska et al. [15] found that Polish adolescents, particularly girls, were among those most frequently reporting attempts to lose weight, with the highest prevalence observed in 15-year-olds (31.8%) compared to younger age groups. Although weight reduction behaviors (WRBs) were more common among girls, a significant upward trend was also observed among Polish boys across all age categories, highlighting a narrowing gender gap in weight-related concerns. Furthermore, in the context of problematic social media use, Polish 11- and 13-year-olds scored above the average for the international sample, this was particularly evident among girls (11-year-old girls: 10% vs. 9% average, 13-year-old girls: 19% vs. 16% average) [23].

The study reported herein represents a continuation both of research conducted within the HBSC network and of the authors' own prior work [24]. Previous studies have explored the associations between screen time behaviors and the prevalence of obesity, adolescents' engagement in physical activity [25], sleep quality [26], and risk-taking behaviors [27]. The authors' own contributions to the field, in turn, have over the past several years examined the relationship between screen time and adolescent well-being, particularly in the context of body image perception [28].

Despite the increasing number of studies addressing the health impact of screen time on adolescents, many aspects of this relationship remain insufficiently investigated. Many existing studies treat screen time as a holistic phenomenon or refer to its different forms without considering usage profiles [2,3,9,12,13]. However, adolescents vary widely not only in how much time they spend on screens, but also in how they use them, whether primarily for gaming, social media, streaming content, or across multiple platforms. Thus, it is essential to investigate whether different screen use profiles, such as gaming-oriented, social media–oriented, or mixed users, differ in their associations with body image perception and WRB. Understanding these distinctions is crucial for developing targeted, evidence-based interventions to support adolescent health.

Therefore, the aim of this study is to analyse screen use profiles among Polish adolescents and their relationship to body image and WRB. In particular, the study seeks to:

– identify different screen-use profiles groups of adolescents who differ in the type and frequency of screen use and determine the prevalence of these profiles in the population, examining their distribution by such demographic and social factors as gender, age, socioeconomic status and place of residence;
– assess the relationship between screen-time profiles and both body image and WRB, with a focus on how different screen time patterns may influence adolescents' self-perceived body size and engagement in dieting.

## MATERIAL AND METHODS

This study was conducted as part of the international HBSC study [24]. During the 2021/2022 school year, in March–June 2022, anonymous computer-assisted online interviews (CAWI) were conducted in school computer labs using an online questionnaire. To ensure geographical diversity, schools were randomly selected from all 16 Polish regions, covering both urban and rural areas. Ultimately, 169 schools (out of 252 invited) participated, resulting in a response rate of 67.1% and refusal rate at an acceptable level of 30.3%. A total of 7111 adolescents participated in the Polish HBSC study. It includes data from students aged 11 years, 13 years and 15 years, which were checked and accepted by the Bergen International Data Bank, in accordance with the HBSC protocol. Additionally, outside the scope of the international protocol, a supplementary sample of 17-year-olds was collected in Poland to gain a broader insight into the health and behavior of older adolescents.

The present study is based on data from 5322 school students across 3 age groups: 13 years old, 15 years old, and 17 years old, corresponding to seventh grade of primary school (K7), and the first and third grades of secondary school (K9 and K11), respectively.

Only adolescents with complete data on the 4 variables relating to sedentary behavior, as well as gender and age group, were included in the study. Data from 11-year-olds were not available for this analysis, as the respective question block was not displayed in the version of the questionnaire completed by this age group.

The study received approval from the Bioethics Committee of the Institute of Mother and Child in Warsaw (No. 51/2021, 2021 June, 24). Participation was voluntary; written parental consent and pupil consent were required. Students had the right to withdraw from the study at any time.

### Screen-time-related behaviors

Screen-time behavior was extracted from responses to 4 different questions [17]: “In your free time: how many hours a day do you spend…”:

– “…playing games on a computer, game console, tablet, smartphone, or smart TV,”
– “…using computer and other electronic devices for social networks for example Instagram, Facebook, Twitter, Snapchat etc.,”
– “…watching TV, DVDs, or videos including internet videos on websites like YouTube etc.,”
– “…looking up for information on the internet, browsing the internet.”

Response categories included: “none at all,” “about half an hour a day,” “about 1 hour a day,” “about 2 hours a day,” “about 3 hours a day,” “about 4 hours a day,” “about 5 hours a day,” “about 6 hours a day,” and “about 7 hours a day or more.” Responses were recoded into numerical values ranging from 0 to 7 (with “half an hour” coded as 0.5), so the average represents the number of hours spent on a given activity. These values are not cumulative, as adolescents often engage in multiple screen-based activities simultaneously.

### Body image

The single-item HBSC body size perception question is as follows: “Do you think your body is…?” with 5 answers ranging from “much too thin” to “much too fat” [24].

### Dieting behavior

Dieting behavior was assessed based on responses to the question: “At present, are you on a diet or doing something else to lose weight?” The response options included: “no, my weight is fine,” “no, but I should lose some weight,” “no, I need to put on weight,” and “yes” [17].

### Body mass index

Self-reported data included information about adolescents' body height and body weight, obtained via the single-item questions from the HBSC questionnaire: “How tall are you with no clothes on?” (reported in cm), and “How much do you weigh with no clothes on?” (reported in kg) [24]. Body mass index (BMI) was calculated as self-reported weight in kg divided by the square of self-reported height in meters. Underweight, normal weight, overweight and obesity were determined using the standardized age- and gen der-specific International Obesity Task Force (IOTF) BMI cut-off points [29].

### Family affluence scale

Family wealth was examined using the revised *Family Affluence Scale* (FAS III), a scale used to measure the material status of families surveyed in HBSC studies of students. It includes 6 items: number of family cars, number of computers in the family, having a bedroom of one's own, number of bathrooms, having a dishwasher in the household, and number of family holidays in the last year [24]. The scale has been validated through both quantitative and qualitative studies, which have also been conducted in Poland. The scale ranges from 0 to 13 pts, with categories of affluence defined as follows: low (0–6 pts), average (7–9 pts), and high (10–13 pts) [30].

### Place of residence

The size of localities was classified based on population thresholds commonly used in national statistics in Poland:

– large city – >100 000 inhabitants,
– small city – 50 000–100 000 inhabitants,
– small town – <50 000 inhabitants,
– rural areas – a settlement classified as a village.

These categories reflect the administrative division and demographic differences recognised by the Statistics Poland (Główny Urząd Statystyczny – GUS) and have been adjusted for clarity in the questionnaire completed by the adolescents [31].

### Statistical analysis

Profiles of screen use were identified using k-means cluster analysis. Solutions ranging 4–6 clusters were tested. The optimal number of clusters was determined using the Elbow Method, by examining changes in the within-cluster sum of squares (WCSS) index. Homogeneity of the clusters was also assessed using ANOVA with Tukey's post hoc test. The 5-cluster solution was selected as optimal, with convergence achieved after 23 iterations, indicating that further iterations no longer altered cluster membership. The 5-cluster solution produced the most diverse groups in terms of the classification variables. It was hypothesised that the sample would include a group of adolescents spending a lot of time on all screen-based activities, a group with generally low screen time, and several groups favouring specific types of activities. The resulting cluster groups were recoded and labelled as follows:

– cluster 1 (C1): moderate users, adolescents with low screen time spent on all activities (gaming, social media use, watching audiovisual content, and internet browsing);
– cluster 2 (C2): game-oriented users, adolescents with elevated screen time spent specifically on gaming (on a computer, console, tablet, smartphone, or TV);
– cluster 3 (C3): social-media-oriented users, adolescents with elevated screen time spent specifically on social networking;
– cluster 4 (C4): intensive game and social media users, adolescents with elevated screen time spent on both gaming and social media;
– cluster 5 (C5): overall intensive users, adolescents with elevated screen time spent on gaming, social media, watching audiovisual content, and browsing the internet.

No cluster was identified that was characterized solely by elevated time spent watching videos or browsing the internet. The percentage of adolescents assigned to each cluster was as follows: C1 – 40.1%, C2 – 21.1%, C3 – 20.3%, C4 – 11.1%, C5 – 7.4%.

Cross-tabulations show the distribution of clusters across social groups, as well as responses to questions concerning body image and dieting within each cluster. The significance of differences between these categorical variables was assessed using the χ^2^ test, and adjusted standardized residuals (Z-score) were also reported. Its absolute value above 1.96 indicated a deviation from the expected distribution, while values above 3 indicated a substantial deviation.

The final stage of the analysis involved estimating 2 multinomial logistic regression models. The first model examined predictors of body image, with the dependent variable categorized as follows: “too thin,” “too fat,” and “about right” (reference group). The second model explored factors associated with responses to a question about whether the participant was dieting, with the dependent variable coded as: “no, but I should lose some weight”; “no, because I need to gain weight”; “yes”; and “no” (reference category). Independent variables included gender, grade, BMI (continuous), cluster membership, categorized FAS III, and place of residence. Results were presented as odds ratios with 95% confidence intervals (CI) for BMI and cluster membership.

Statistical analyses were performed on IBM SPSS Statistics for Windows, v. 28.0 (IBM Corp., 2021).

## RESULTS

### Sample characteristics

The analysis included data from 5322 adolescents (45.5% of them boys, and 42.0% living in rural areas) aged 13 years (N = 1545), 15 years (N = 2104) and 17 years (N = 1673). The age of the participants was mean (M) ± standard deviation (SD) 15.46±1.45 years. More than half were adolescents from families of average affluence (51.7%), while 27.1% reported low affluence and 21.2% high affluence. The family affluence score was M±SD 7.77±2.18. Over-weight or obesity was present in 19.2% of adolescents and the BMI Z-score was M±SD 0.09±1.19. Nearly half of all participants reported perceiving themselves as “a bit too fat” or “much too fat” (45.6%), while 18% considered themselves “a bit too thin” or “much too thin”. Nearly 1 in 5 adolescents (18%) reported being on a diet or taking other measures to lose weight (18%), while a quarter (24.8%) expressed intentions to lose weight. The characteristics of the sample are presented in Table 1.

**Table 1:** Sample characteristics, Health Behaviour in School-age Children (HBSC) study, Poland, 2021–2022

Variable	Participants (N = 5322)	
	n	%
Gender		
boys	2424	45.5
girls	2898	54.5
Grade		
K7	1545	29.0
K9	2104	39.5
K11	1673	31.4
*Family Affluence Scale* (FAS III)		
low	1417	27.1
average	2708	51.7
high	1109	21.2
Domicile		
large city	879	16.6
small city	672	12.7
small town	1525	28.7
rural area	2229	42.0
BMI		
thinness	634	13.0
normal weight	3309	67.8
overweight/obesity	936	19.2
Body image^a^		
“much too thin”	225	4.4
“a bit too thin”	698	13.6
“about right”	1877	36.5
“a bit too fat”	1782	34.6
“much too fat”	567	11.0
Dieting		
“no, my weight is fine”	2267	42.9
“no, but I should lose some weight”	1310	24.8
“no, because I need to put on weight”	758	14.3
“yes”	954	18.0

K7 – the seventh grade of primary school; K9 – the first grade of secondary school, K11 – the third grade of secondary school.

a In subsequent analyses, the marginal categories were combined.

### Screen time by type of activity

**Figure 1:**
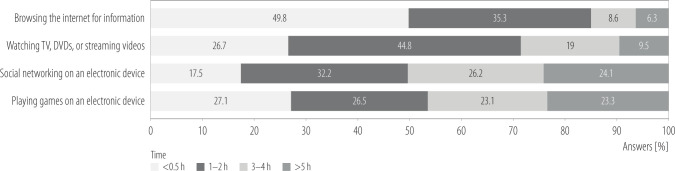
Time spent by adolescents on different forms of screen-based activities, Health Behaviour in School-aged Children (HBSC) study, Poland, 2021–2022

The data presented in Figure 1 indicate that the shortest screen time was reported for browsing the internet for information; nearly half of adolescents (49.8%) spent ≤0.5 h/day on this activity. In contrast, the longest durations were reported for social media use and playing games, with 24.1% and 23.3% of adolescents, respectively, spending ≥5 h/day on these activities. Watching audiovisual content was moderately common, with 44.8% reporting 1–2 h/day. The average time spent on screen time-related activities across the identified clusters was presented in Table 2.

**Table 2:** The average time spent on screen time-related activities across the identified clusters, Health Behaviour in School-aged Children (HBSC) study, Poland, 2021–2022

Cluster	Activity time [h/day] (M±SD)			
	playing games on an electronic device	social networking on an electronic device	watching TV, DVDs, or streaming videos	browsing the internet for information
Moderate users (C1)	1.17±1.00	1.46±0.97	1.16±1.02	0.82±0.84
Game-oriented users (C2)	4.59±1.34	1.53±1.01	2.31±1.63	1.06±1.08
Social-media-oriented users (C3)	1.00±1.08	4.95±1.30	2.01±1.59	1.41±1.36
Intensive game and social media users (C4)	5.62±1.21	5.33±1.30	1.85±1.39	1.25±1.10
Overall intensive users (C5)	5.53±1.93	5.73±1.69	5.24±1.94	4.93±2.04
Total	2.67±2.30	2.93±2.16	1.95±1.75	1.34±1.56

The analysis revealed significant differences in screen time across clusters (p < 0.001). Overall intensive users (C5) and those combining gaming and social media engagement (C4) spent the most time on games and social media, while moderate users (C1) and game-preferring (C2) users reported the lowest values. Watching audiovisual content was highest among overall intensive users (C5), whereas moderate users (C1) and gaming-focused users (C2) spent the least amount of time in this way. Browsing the internet was most time-consuming for the overall intensive-use cluster (C5), whilst moderate users (C1) showed the lowest engagement.

### Sociodemographic, BMI, body image and dieting behavior characteristics of adolescents in relation to screen-time clusters

The results of cluster distribution across socio-demographic groups can be seen in Table 3. The gaming-oriented cluster (C2) was nearly 3 times more prevalent among boys than among girls, while girls were over twice as likely as boys to belong to the social-media-oriented cluster (C3). With age, the proportion of the gaming-oriented cluster (C2) decreased, while that of the social-media-oriented cluster (C3) increased. In the first grade of secondary school, the over-representation of C4 is particularly noticeable, and in the third grade of secondary school, an excess of intensive use of social media alone (C3) stands out. Socioeconomic status showed minor variation, with the lowest-income group having a slightly higher representation in the overall intensive-use cluster (C5). Adolescents from large cities were more frequently in the moderate-use cluster (C1), while those from rural areas had a higher share in the gaming cluster (C2).

**Table 3:** Cluster distribution across socio-demographic groups, Health Behaviour in School-aged Children (HBSC) study, Poland, 2021–2022

Variable	C1 (N = 2138)		C2 (N = 1121)		C3 (N = 1078)		C4 (N = 593)		C5 (N = 392)		p
	%	Z	%	Z	%	Z	%	Z	%	Z	
Total	40.1		21.1		20.3		11.1		7.4		
Gender											<0.001
boys	41.3	15.0	31.0	16.2	10.4	–16.4	8.8	–4.9	8.5	3.0	
girls	39.3	–1.5	12.8	–16.2	28.5	16.4	13.1	4.9	6.4	–3.0	
Grade											<0.001
K7	43.6	3.3	23.8	3.2	13.7	–7.9	10.7	–0.6	8.1	1.3	
K9	36.1	–4.9	22.0	1.4	21.7	2.1	13.6	4.6	6.7	–1.6	
K11	42.1	2.0	17.3	–4.5	24.5	5.2	8.4	–4.3	7.6	0.4	
*Family Affluence Scale* (FAS III)											0.006
low	39.6	0.4	20.7	–0.4	19.1	–1.2	11.4	0.3	9.2	3.0	
average	39.7	–0.5	22.3	2.4	20.8	1.1	11.0	–0.6	6.1	–3.7	
high	41.4	1.0	18.5	–2.4	20.2	0.0	11.6	0.4	8.3	1.3	
Domicile											0.014
large city	42.3	1.4	19.8	–1.0	18.4	–1.5	11.1	0.0	8.3	1.2	
small city	37.2	–1.7	21.4	0.3	19.6	–0.5	12.6	1.3	9.1	1.8	
small town	37.8	–2.2	23.0	2.2	19.9	–0.5	12.1	0.15	7.2	–0.3	
rural area	41.8	2.1	20.1	–1.5	21.6	1.9	10.0	–2.2	6.6	1.8	

C1 – moderate users, C2 – game-oriented users, C3 – social-media-oriented users, C4 – intensive game and social media users, C5 – overall intensive users.

K7 – the seventh grade of primary school; K9 – the first grade of secondary school 9, K11 – the third grade of secondary school.

Z – adjusted standardized residual.

The results of distribution of BMI categories, body image, and dieting in clusters can be seen in Table 4. The clusters differed significantly in terms of in BMI, body size perception, and dieting behaviors (all p < 0.001). The highest prevalence of overweight/obesity was observed in the overall intensive-use cluster (C5), while normal weight was most common in the social-media-oriented cluster (C3). Adolescents in C3, C4, and C5 more frequently perceived themselves as “too fat,” aligning with a higher tendency to express a desire to lose weight. Dieting was most prevalent in C3 and C5, whereas the belief that their weight was “about right” was most common in C1.

**Table 4:** Distribution of BMI categories, body image, and dieting in clusters, Health Behaviour in School-aged Children (HBSC) study, Poland, 2021–2022

Variable	C1 (N = 2138)		C2 (N = 1121)		C3 (N = 1078)		C4 (N = 593)		C5 (N = 392)		p
	%	Z	%	Z	%	Z	%	Z	%	Z	
BMI											<0.001
thinness	12.9	–0.1	10.9	–2.2	14.0	1.1	15.0	1.5	13.3	0.2	
normal weight	69.8	2.4	64.6	–2.5	71.8	3.0	65.9	–1.0	57.8	–4.1	
overweight/obesity	17.3	–2.7	24.5	4.9	14.1	–4.5	19.1	–0.1	28.9	4.7	
Body image											<0.001
“too thin”	17.7	–0.3	21.6	3.5	15.7	–2.1	16.2	–1.1	17.1	–0.4	
“about right”	42.4	7.2	35.6	–0.6	31.4	–3.8	30.3	–3.2	29.7	–2.8	
“too fat”	39.9	–6.8	42.8	–2.1	52.9	5.3	53.4	4.0	53.2	3.1	
Diet											<0.001
“no, my weight is fine”	49.2	7.6	41.6	–0.9	37.6	–3.9	36.5	–3.3	36.2	–2.8	
“no, but I should lose some weight”	20.9	–5.3	24.3	–0.4	28.9	3.5	30.9	3.7	26.4	0.8	
“no, because I need to put on weight”	13.2	–1.8	18.0	4.0	13.0	–1.4	13.4	–0.7	14.6	0.2	
“yes”	16.6	–2.2	16.1	–1.9	20.5	2.3	19.2	0.8	22.8	2.6	

Abbreviations as in Table 3.

### Multinomial logistic regression: body image and dieting behaviors according to screen use profiles and BMI Z-score

Looking at the first model (Table 5), a higher BMI Z-score was found to be associated with lower likelihood of perceiving oneself as “too thin” (OR = 0.401, 95% CI: 0.362–0.444) and increased odds of feeling “too fat” (OR = 2.358, 95% CI: 2.182–2.548). Among screen time clusters, “intensive game and social media users” (C4) showed higher odds of perceiving themselves as “too thin” (OR = 1.395, 95% CI: 1.016–1.914) compared with the reference group. For the “too fat” perception, all heightened-use clusters showed increased odds, with the strongest effect observed in “overall intensive users” (OR = 2.064, 95% CI: 1.534–2.777).

**Table 5:** Multinomial logistic regression models for the body image variable, with “about right” and diet as the reference group–association with clusters and BMI Z-score, adjusted for gender, grade, *Family Affluence Scale* (FAS III), place of residence, Health Behaviour in School-aged Children (HBSC) study, Poland, 2021–2022

Variable	β	SE	p	OR (95% CI)
Model 1: Body image				
too thin				
constant	–1.834	0.159	0.000	
BMI Z-score (continuous)	–0.914	0.052	0.000	0.401 (0.362–0.444)
screen time cluster				
C2	0.154	0.121	0.204	1.166 (0.920–1.477)
C3	0.228	0.133	0.087	1.256 (0.967–1.630)
C4	0.333	0.161	0.039	1.395 (1.016–1.914)
C5	0.062	0.207	0.766	1.064 (0.709–1.596)
C1 (ref.)				1.000
too fat				
constant	0.267	0.116	0.021	
BMI Z-score (cont.)	0.858	0.040	0.000	2.358 (2.182–2.548)
screen time cluster				
C2	0.360	0.099	0.000	1.433 (1.181–1.739)
C3	0.471	0.098	0.000	1.602 (1.322–1.941)
C4	0.544	0.121	0.000	1.723 (1.358–2.184)
C5	0.725	0.151	0.000	2.064 (1.534-2.777)
C1 (ref.)				1.000
Model 2: Diet				
“no, but I should lose some weight”				
constant	–0.488	0.134	0.000	
BMI Z-score (continuous)	1.129	0.047	0.000	3.092 (2.821–3.389)
screen time cluster				
C2	0.361	0.113	0.001	1.434 (1.149–1.792)
C3	0.542	0.110	0.000	1.720 (1.385–2.135)
C4	0.675	0.135	0.000	1.964 (1.509–2.557)
C5	0.464	0.169	0.006	1.590 (1.142–2.213)
C1 (ref.)				1.000
“no, because I need to put on weight”				
constant	–2.408	0.171	0.000	
BMI Z-score (continuous)	–1.025	0.056	0.000	0.359 (0.321–0.401)
screen time cluster				
C2	0.328	0.125	0.009	1.388 (1.086–1.774)
C3	0.359	0.137	0.009	1.431 (1.094–1.873)
C4	0.305	0.170	0.072	1.357 (0.973–1.892)
C5	0.143	0.210	0.496	1.154 (0.765–1.740)
C1 (ref.)				1.000
“yes”				
constant	–0.442	0.139	0.001	
BMI Z-score (continuous)	0.980	0.049	0.000	2.665 (2.421–2.935)
screen time cluster				
C2	0.243	0.123	0.049	1.274 (1.001–1.622)
C3	0.280	0.119	0.018	1.324 (1.048–1.671)
C4	0.382	0.148	0.010	1.465 (1.097–1.956)
C5	0.594	0.172	0.001	1.810 (1.293–2.535)
C1 (ref.)				1.000

Abbreviations as in Table 3.

Looking at the second model (Table 5), a higher BMI Z-score increased the odds of expressing a desire to lose weight (OR = 3.092, 95% CI: 2.821–3.389) and being on a diet (OR = 2.665, 95% CI: 2.421–2.935) while reducing the likelihood of reporting a need to gain weight (OR = 0.359, 95% CI: 0.321–0.401). Compared to moderate users, all intensive-use clusters (C2–C5) showed elevated odds of intending to lose weight, with the highest effect among “intensive game and social media users” (C4) (OR = 1.964, 95% CI: 1.509–2.557). For active dieting, the strongest association was observed among “intensive users” (C5) (OR = 1.810, 95% CI: 1.293–2.535). Those in the gaming (C2) and social media (C3) clusters were also more likely to express a need to gain weight, though the effects were weaker.

## DISCUSSION

The study presented herein analysed data on 5322 Polish students aged 13–17 years to explore associations between various types of screen-time activities and Polish adolescents' body image perception, BMI, and dieting-related behaviors. Generally, the study confirmed strong associations between screen time profiles and Polish adolescents' body image and dieting behaviors. More specifically, the authors found that heightened screen use (especially in the intensive-use C4 and C5 clusters) is linked to higher BMI, greater body dissatisfaction, and increased prevalence of dieting. Moreover, it was found that BMI Z-score independently predicts both body image distortion and dieting behavior.

### Identifying different screen use profiles

The clustering analysis in the study identified 5 distinct screen-use profiles, with a large majority of adolescents grouped in the moderate use (C1, 40.1%), game-oriented (C2, 21.1%), and social-media-oriented (C3, 20.3%) clusters. Notably, only a small proportion of youth belonged to the 2 more intensive use profiles (C4 and C5, 8.5% combined). This distribution aligns with the results reported by da Costa et al. [32], who also found a dominant low or singleplatform usage (with “Low ST” and “High Cellphone” groups together accounting for 64% of all participants) and only a minority engaging broadly across multiple screen types (the “High ST” group, 20%). Similarly, the systematic review by Ferrar et al. [33] emphasized the recurring identification of moderate and single-behavior clusters such as sport-, screen-, or low-engaged profiles, as well as the utility of cluster analysis for capturing complex behavioral patterns. Together, these findings underscore the importance of tailoring interventions to the dominant, yet more moderate, user profiles, while not overlooking the potential health risks faced by smaller groups of highly intensive users.

This is further supported by Peiró-Velert et al. [34], who combined clustering techniques with self-organizing maps to explore associations between screen time, sleep, and academic performance. Their results, showing that higher computer use often coincided with increased TV and mobile phone use, mirror the authors' own observation that audiovisual content consumption was highest among the most intensive screen users (C5). Given the growing use of wearable technologies among youth, future studies could enhance accuracy by incorporating objective monitoring methods, as suggested by Prince et al. [35] and Mazur et al. [36], which may also help boost adolescent engagement in research.

These findings, in summary, highlight the complexity of adolescents' screen-use patterns and the importance of ongoing monitoring. As the HBSC survey is repeated every 4 years and allows for international comparisons, future research could investigate whether the screen-use profiles identified in this study remain consistent over time and across cultural contexts.

### Screen time patterns according to demographic and social factors

Our findings indicate that screen time usage profiles are not uniformly distributed across key demographic and social groups. The cluster analysis revealed clear differences in screen use according to gender, age, and place of residence. As expected, adolescents' screen use profiles varied markedly between boys and girls: boys were overrepresented in clusters dominated by gaming activities (C2), whereas girls were more likely to belong to clusters centred around social media use (C3) and intensive multi-platform engagement (C5). These sex-based patterns are consistent with broader trends in digital media use, reflecting distinct interests and motivations for screen engagement among adolescents [35,37,38].

An age-related gradient was also evident. Younger adolescents were more commonly associated with gaming-oriented profiles, whereas older students tended to cluster into groups marked by high levels of social media engagement. This age-related transition is in line with prior research showing a decline in video gaming and a rise in social media use with age, likely reflecting both developmental shifts in interests and the increasing importance of peer interaction and self-expression in later adolescence [39,40].

With regard to place of residence, this study results showed that young people from large cities were more frequently represented in intensive screen use clusters. This observation may reflect greater availability of digital infrastructure, higher digital literacy, and stronger exposure to urban cultural trends related to technology, appearance, and lifestyle [41]. Interestingly, this finding contrasts with results from a large-scale German study involving over 12 000 children and adolescents aged 4–17 years, which reported that screen time, particularly computer and gaming use, increased across all types of areas except cities, with the sharpest rise observed in rural regions. Notably, the increase was especially pronounced among girls [42], suggesting that urban–rural patterns in screen behavior may vary significantly between countries and reflect broader sociocultural and infrastructural differences.

Socioeconomic status (SES) was another important factor associated with screen time profiles. In the German study, SES, as measured by the FAS III, showed a clear link with screen-related behaviors, which aligns with findings from other countries. For instance, Finnish research indicated that lower parental SES was associated with higher levels of screen exposure in both genders, but particularly among girls, for whom it may constitute a specific risk factor for excessive media use [43]. These findings support growing evidence that SES not only shapes access to digital tools but also influences the patterns and purposes of their use [44]. Taken together, these data suggest that effective intervention strategies should account for not only gender and age, but also the broader social and environmental contexts in which adolescents engage with screen-based media.

### Body image and weight reduction behavior across the screen time profiles

This study demonstrates a strong association between screen time patterns and both body image perception and dieting behaviors among adolescents. Youth in the heightened-use clusters especially the social-media-oriented (C3), gaming and social media-oriented (C4), and fully intensive (C5) groups, were more likely to perceive themselves as “too fat” and attempt weight loss. Intensive gaming and social media users (C4) also more often perceived themselves as “too thin.” The strongest link with the intention to lose weight was observed in cluster C4, while active dieting was most prevalent in overall intensive users (C5). Additionally, adolescents in the gaming (C2) and social media (C3) clusters more frequently expressed a desire to gain weight. Although higher BMI remained the primary predictor of weight-related perceptions, screen time independently intensified body dissatisfaction and dieting behaviors, even after adjusting for BMI and sociodemographic variables.

Excessive overall screen time, embracing social media use, television, internet browsing, and gaming, can significantly distort adolescents' body image, fostering beliefs of being “too fat” even without excess body weight [38]. Exposure to idealized images, reinforced by social comparison, sedentary behavior, algorithm-driven content, and idealized avatars, intensifies appearance concerns. This may lead adolescents to increased body monitoring, pressure to conform to beauty ideals, and engagement in dieting behaviors, even at a healthy weight [15].

The findings presented in the authors' article are consistent with a growing body of international research indicating that prolonged exposure to screen-based media, particularly highly visual social media (HVSM) such as Instagram, TikTok, and Snapchat, is associated with increased body dissatisfaction, internalization of unrealistic appearance ideals, and engagement in disordered eating behaviors [45]. Findings from Iceland also indicate that greater screen time is linked to a deterioration in body image among adolescent girls, as evidenced by lower body image scores observed both cross-sectionally and longitudinally between ages 15–17 years, emphasizing the persistent negative influence of digital media exposure on body image development [46,47]. Social comparisons, as common on social media especially with close friends, play a key role in body dissatisfaction among adolescent girls. Scully et al. [48] found that time spent on appearance-based comparisons online significantly predicted poor body image, partly through internalizing the thin ideal, even after adjusting for age and self-esteem.

Even after adjusting for BMI and sociodemographic factors, intensive screen use remained strongly associated with negative body perceptions and dieting behaviors which may also supports the notion that adolescents' body image is shaped not only by actual weight status but also by exposure to media-driven appearance ideals. These results are consistent with data from the Polish HBSC study, which revealed widespread misperception of body weight among adolescents, particularly among girls, indicating a high vulnerability to distorted body image in this group [49].

These findings also indicate that higher level of screen time is associated with an increased likelihood of adolescents engaging in weight loss behaviors. The relationship may reflect the pervasive influence of digital content promoting thinness and dieting, particularly on social media platforms frequently used by youth. Supporting the results, a large international study found that higher screen time, especially on platforms like Twitter, was linked to increased attempts to lose weight among adolescents [50]. Consistent with this, a longitudinal study of over 10 000 youths showed that more daily screen time predicted a higher risk of eating disorder symptoms 2 years later, particularly in those with problematic social media and mobile phone use [51].

### Limitations

This study has a number of limitations that should be considered. The use of self-reported data may have introduced bias due to social desirability or recall errors, potentially affecting the accuracy of reported screen time and body-related perceptions. As a cross-sectional study, the design does not allow for causal inferences regarding the relationship between screen use and health outcomes. Additionally, the absence of objective screen time measurements (e.g., device or app tracking) limits the precision of exposure assessment. Body image and dieting behaviors were assessed using single-item indicators, which may not fully reflect their complexity. Lastly, as the study was conducted exclusively in Poland, the findings may not be generalisable to adolescents in different cultural or media environments.

## CONCLUSIONS

The present study has found that intensive screen use, particularly involving social media and gaming activities, is strongly associated with higher BMI, greater body dissatisfaction, and an increased prevalence of dieting behaviors among adolescents. Of particular significance is the finding that screen time exerted an independent effect on body image and dieting, even after adjusting for BMI and sociodemographic factors. It is evident that patterns of screen use vary according to gender, age, and place of residence. This observation underscores the necessity to consider demographic and social contexts when addressing adolescent health behaviors.

The findings emphasise the significance of promoting media literacy among adolescents as a means of mitigating the internalisation of unrealistic appearance ideals that are reinforced by digital media [52]. Prevention strategies should be tailored to vulnerable groups, with a particular focus on girls, older adolescents, and urban youth. The involvement of parents, educators, and healthcare professionals is crucial in fostering healthier screen use habits and supporting positive body image development. It is recommended that future public health interventions incorporate digital behavior monitoring and strive to tackle the wider social determinants of excessive screen use.
